# The Current Status and Promotional Strategies for Cloud Migration of Hospital Information Systems in China: Strengths, Weaknesses, Opportunities, and Threats Analysis

**DOI:** 10.2196/52080

**Published:** 2024-02-05

**Authors:** Jian Xu

**Affiliations:** 1 Department of Health Policy Beijing Municipal Health Big Data and Policy Research Center Beijing China

**Keywords:** hospital information system, HIS, cloud computing, cloud migration, Strengths, Weaknesses, Opportunities, and Threats analysis

## Abstract

**Background:**

In the 21st century, Chinese hospitals have witnessed innovative medical business models, such as online diagnosis and treatment, cross-regional multidepartment consultation, and real-time sharing of medical test results, that surpass traditional hospital information systems (HISs). The introduction of cloud computing provides an excellent opportunity for hospitals to address these challenges. However, there is currently no comprehensive research assessing the cloud migration of HISs in China. This lack may hinder the widespread adoption and secure implementation of cloud computing in hospitals.

**Objective:**

The objective of this study is to comprehensively assess external and internal factors influencing the cloud migration of HISs in China and propose promotional strategies.

**Methods:**

Academic articles from January 1, 2007, to February 21, 2023, on the topic were searched in PubMed and HuiyiMd databases, and relevant documents such as national policy documents, white papers, and survey reports were collected from authoritative sources for analysis. A systematic assessment of factors influencing cloud migration of HISs in China was conducted by combining a Strengths, Weaknesses, Opportunities, and Threats (SWOT) analysis and literature review methods. Then, various promotional strategies based on different combinations of external and internal factors were proposed.

**Results:**

After conducting a thorough search and review, this study included 94 academic articles and 37 relevant documents. The analysis of these documents reveals the increasing application of and research on cloud computing in Chinese hospitals, and that it has expanded to 22 disciplinary domains. However, more than half (n=49, 52%) of the documents primarily focused on task-specific cloud-based systems in hospitals, while only 22% (n=21 articles) discussed integrated cloud platforms shared across the entire hospital, medical alliance, or region. The SWOT analysis showed that cloud computing adoption in Chinese hospitals benefits from policy support, capital investment, and social demand for new technology. However, it also faces threats like loss of digital sovereignty, supplier competition, cyber risks, and insufficient supervision. Factors driving cloud migration for HISs include medical big data analytics and use, interdisciplinary collaboration, health-centered medical service provision, and successful cases. Barriers include system complexity, security threats, lack of strategic planning and resource allocation, relevant personnel shortages, and inadequate investment. This study proposes 4 promotional strategies: encouraging more hospitals to migrate, enhancing hospitals’ capabilities for migration, establishing a provincial-level unified medical hybrid multi-cloud platform, strengthening legal frameworks, and providing robust technical support.

**Conclusions:**

Cloud computing is an innovative technology that has gained significant attention from both the Chinese government and the global community. In order to effectively support the rapid growth of a novel, health-centered medical industry, it is imperative for Chinese health authorities and hospitals to seize this opportunity by implementing comprehensive strategies aimed at encouraging hospitals to migrate their HISs to the cloud.

## Introduction

In the 21st century, innovative business models have emerged in Chinese hospitals, such as online diagnosis and treatment, cross-regional multidepartment consultation, real-time sharing of medical test results, and continuous public health surveillance. However, most hospitals still rely on traditional hospital information systems (HISs) designed for in-hospital management that are inadequate to support the development of these new business models [1]. Cloud computing has emerged as a promising global information technology recognized as a new infrastructure for future economic growth [2,3]. Since 2010, it has also been prioritized by the Chinese government as a “national strategic emerging industry” [4]. The adoption of cloud computing technologies can significantly reduce hospitals’ costs associated with system construction and maintenance [5], expand medical services to partner institutions or patients outside the hospital [6], provide more secure network protection than self-built data centers [7], and facilitate large-scale collection and analysis of clinical data essential for scientific clinical decision-making [8]. Based on these advantages, there has been a surge in China’s medical cloud service market and application research in recent years [9].

However, despite the increased attention given to cloud computing in various disciplinary domains such as disease monitoring, health surveillance, and clinical diagnosis, there is a lack of research on the cloud migration of HISs. A comprehensive review of the PubMed and HuiyiMd databases only yielded 3 relevant studies: an Iranian study that identified key driving factors for hospitals adopting cloud computing [10], a Greek study that proposed a method for migrating clinical and laboratory data based on local hospital conditions [11], and an American study that focused on essential considerations for chief financial officers before venturing into the cloud [12]. However, none of these studies have adequately addressed the aforementioned issue. Without conducting prior assessments, hospitals may struggle to fully comprehend the external environment, internal conditions, and potential opportunities and risks, thus failing to ensure prudent decision-making. Blindly following trends could pose significant threats to the security, operational efficiency, and maintenance costs of already deployed cloud-based information systems and existing hospital networks [13]. Therefore, this study aims to systematically assess factors influencing the cloud migration of HISs in China, identify associated challenges, and propose corresponding strategies for advancement. It can assist hospitals in gaining a comprehensive understanding of this work while safely implementing their cloud-based medical services. Additionally, it serves as a foundation for formulating policies aligned with Chinese hospital informatization development in the new era by health authorities while being referenced by other countries or regions facing similar challenges.

## Methods

### Information Sources

The primary data source for this study was obtained from literature databases to understand the practical applications of cloud technology in Chinese hospitals. The articles published between January 1, 2007, and February 21, 2023, were selected from MEDLINE (accessible through PubMed) and HuiyiMd (accessible through the Huiyi Medical Literature Express Service System).

In order to overcome the inherent limitations of academic articles, this study augmented a wealth of pertinent internal and external environmental information by extensively consulting authoritative sources such as government agencies, industry organizations, academic institutions, and market research companies. These sources of information included national policies, action plans, white papers, implementation guidelines, survey reports, and statistical data from the past 10 years.

### Search Strategies

The search strategy for PubMed: (((cloud [Title/Abstract]) OR (cloud-based [Title/Abstract])) AND (hospital [Title/Abstract]) AND (“2007/01/01” [Date-Publication]: “2023/02/21” [Date-Publication])). The search strategy for HuiyiMd: ([TI] (cloud AND hospital) OR [AB] (cloud AND hospital) OR [MH] (cloud AND hospital)) AND ([PY]>=2007). The search strategy for authoritative sources involves entering the keywords “hospital” AND “cloud” in the search box on websites.

### Inclusion and Exclusion Criteria

Based on specified inclusion and exclusion criteria (Textbox 1), irrelevant articles or those covering the same topic from the same institution were excluded. Subsequently, an Excel (Microsoft) spreadsheet (Multimedia Appendix 1) and a reference list (Multimedia Appendix 2 [1,2,6,8,14-26]) were generated for literature review and Strengths, Weaknesses, Opportunities, and Threats (SWOT) analysis.

**Inclusion and exclusion criteria for literature review.**

**Inclusion criteria**

- Article type: fully retrievable
- Language: English, Chinese
- Nationality of the first author: Chinese (including Hong Kong and Taiwan)
- Article topic: the research, development, and application of cloud technology in Chinese hospitals
- Publication date: from January 1, 2007, to February 21, 2023

**Exclusion criteria**

- Article type: nonretrievable
- Language: other languages
- Nationality of the first author: other countries
- Article topic: other topics
- Publication date: before January 1, 2007; after February 21, 2023

### Information Extraction

The accessible articles were assessed based on the following criteria: title, authors, first author, first author affiliation, publication year, journal name, digital object identifier (DOI), PubMed unique identifier (PMID), first author nationality, abstract, and conclusion. Furthermore, the positive and negative impacts, research methods, disciplinary domains, cloud service models, and institutional affiliations were taken into account for further in-depth analysis purposes. The findings were documented and statistically analyzed in Excel.

### Analysis Methods

The SWOT analysis is a systematic assessment of strengths (S), weaknesses (W), opportunities (O), threats (T), and other factors that influence a specific topic, objectively describing the current situation of an organization or enterprise and formulating corresponding strategies [14]. It is widely used in strategic decision-making and competitor analysis within organizations or businesses due to its ability to simplify complex problems into essential issues, enabling more focused problem-solving. This study uses the SWOT method to assess the factors impacting China’s cloud migration of HISs and proposes promotional strategies.

## Results

### Literature Review

#### Identification Process

**Figure 1:**
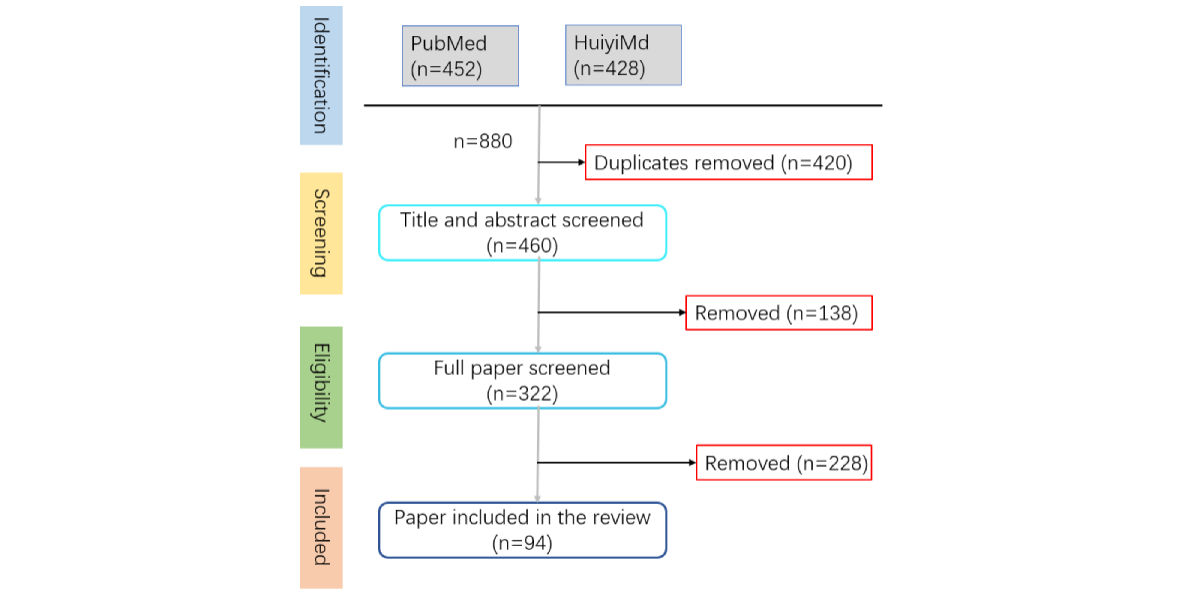
Flow diagram for the identification process.

The identification process in this study consists of four steps (Figure 1): (1) A total of 880 articles were retrieved from PubMed and HuiyiMd databases. (2) The search results were amalgamated, resulting in 460 deduplicated articles. (3) Screening the titles and abstracts eliminated 138 irrelevant articles based on the exclusion criteria. (4) The full text of the remaining articles was meticulously examined against predefined inclusion and exclusion criteria, resulting in a final selection of 94 relevant articles.

#### Comprehensive Description of the Literature

##### Research and Application of Cloud Technology in Hospitals Has Grown Rapidly and Continuously Expanded in Disciplinary Domains

**Figure 2:**
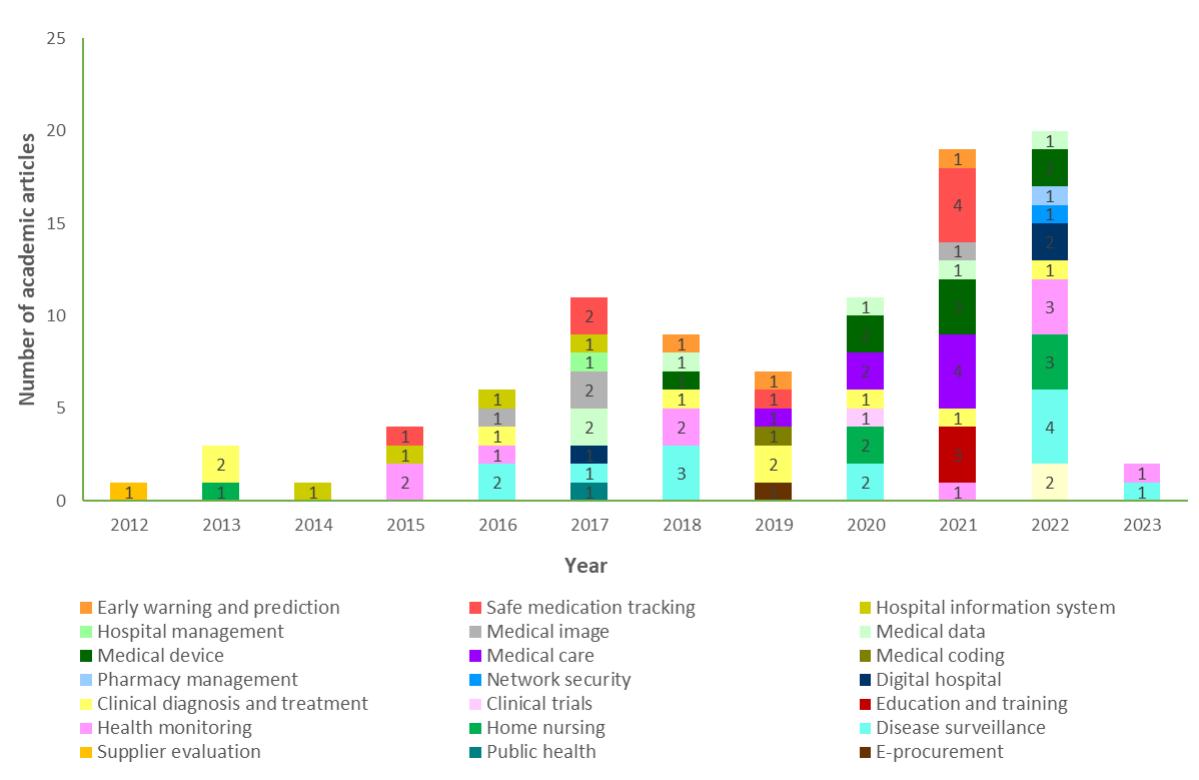
Time distribution of disciplinary domains involved in academic articles.

In terms of time line, there was a gradual step-like increase in the number of articles starting in 2012 and reaching its peak at 20 articles in 2022. The compound annual growth rate (CAGR) was approximately 35%, highlighting the escalating quantity of research into and application of cloud technology in hospitals, as shown in Figure 2. The number of disciplines involved has increased from 1 in 2012 to 10 in 2022, encompassing a total of 22 domains. Specifically, research and application are predominantly observed in the domains of disease monitoring, health surveillance, clinical diagnosis and treatment, safe medication tracking, and medical devices, constituting 51% (48/94) of the overall distribution.

##### Implementation of Cloud Technology Can Yield Favorable Outcomes for Hospitals to a Certain Extent

The analysis of 94 articles identified 3 categories and 9 research methods (Table 1). The “technology” category was the most prevalent, with 47 (50%) articles focusing on information systems, cloud platforms, and associated technologies. The “experience” category followed closely, with 40 (43%) articles, primarily validating the performance of or applying cloud-based information systems through empirical research, case-control studies, experience sharing, and cohort studies. Finally, the “literature” category consisted of only 7 literature reviews on this subject matter. The consistent findings of these studies demonstrate the implementation of cloud technology in hospitals can yield positive impact to some extent, such as enhancing precision in management practices, expanding disease monitoring capabilities, reducing workload for medical personnel, and providing convenient and cost-effective health care services for patients. However, 5% (n=5) of the articles also acknowledged certain negative impacts, such as underdevelopment of digital methods in hospitals, cybersecurity risks, and low satisfaction rates among physicians and community pharmacists.

**Table 1:** The correlation between research methods used in academic articles and the institutional affiliations of their first authors.

Research methods		Hospitals, n (%)	Universities or colleges, n (%)	Associations or companies, n (%)
**Technology**				
	System research and development	13 (14)	14 (15)	N/A^a^
	Cloud platform construction	5 (5)	5 (5)	N/A
	Technical research	2 (2)	8 (9)	N/A
**Experience**				
	Empirical research	14 (15)	7 (7)	1 (1)
	Case control study	11 (12)	2 (2)	N/A
	Summary of experience	3 (3)	N/A	N/A
	Cohort study	2 (2)	N/A	N/A
**Literature**				
	Retrospective study	4 (4)	2 (2)	N/A
	Standard study	N/A	1 (1)	N/A

^a^N/A: not applicable.

##### More Than Half of the Studies Focused on Task-Specific Cloud-Based Systems, While Only 1 in 5 Addressed Integrated Cloud Platforms

**Figure 3:**
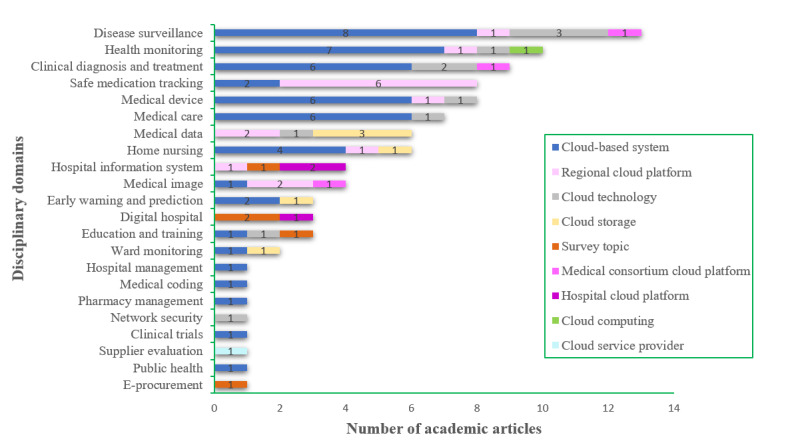
The cross-distribution of cloud service models and disciplinary domains covered in academic articles.

Out of the 94 articles analyzed, the majority (n=49, 52%) focused on task-specific cloud-based systems in hospitals. In contrast, only 21 (22%) articles discussed or developed integrated cloud platforms for sharing within a region, medical alliance, or hospital. Furthermore, as shown in Figure 3, 67% (n=33) of task-specific cloud-based systems were used in patient-related domains such as disease monitoring, health surveillance, clinical diagnosis and treatment, medical care, and medical devices. A total of 15 regional cloud platforms (16%) were commonly used for safe medication tracking, data storage, and medical imaging. A total of 3 medical alliance cloud platforms (3%) found use in disease treatment-related domains such as disease monitoring, clinical diagnosis, and treatment along with medical imaging. A total of 3 hospital cloud platforms (3%) primarily originated from digital hospitals or HIS upgrades.

##### More Supplementary Materials Were Collected to Support the SWOT Analysis for This Study

Because the literature review provided insufficient information to support the analysis of internal and external factors for this study, supplementary materials were collected, including national policies, action plans, white papers, implementation guidelines, research reports, and statistical reports from authoritative websites, such as government agencies, industry organizations, academic institutions, and market research companies. In total, 37 supplementary documents were included in the SWOT analysis: 4 policy papers, 11 industry reports, 15 academic articles, 2 dissertations, 2 official bulletins, and 3 news articles. All of them were recorded in an Excel file (Multimedia Appendix 2).

### SWOT Analysis

#### External Opportunities (O)

##### Politics: Governments Worldwide Prioritize Cloud Technology and Have Implemented Supportive Policies

The United States introduced the Cloud First policy [2] and the CLOUD Act [15]. Similarly, the European Union aims for digital sovereignty through initiatives like the Gaia-X Association and the EU Cloud Computing Strategy [27]. China also prioritized cloud computing as a “national strategic emerging industry” in 2010 and implemented policies to promote its adoption by the government and businesses [28]. Consequently, China has rapidly developed in this field and ranks among global leaders [29].

##### Economy: Both Nations and Enterprises Have Made Substantial Capital Investments to Foster Its Development

According to Gartner’s data from 2011 to 2022, the market scale increased from US $95.24 billion to US $491 billion with a CAGR exceeding 16% [30]. The global medical cloud computing market reached US $39.4 billion, with Asia-Pacific exhibiting the fastest growth rate at 22% per year; China and India are significant contributors to this expansion [9]. In China, the market size has surged from less than US $270 million in 2011 to US $66.91 billion in 2022, with a consistent CAGR surpassing 40%, and will exceed US $150 billion by 2025 [30].

##### Social: Online Health Care Has Become a Norm in Modern Life

According to the National Telemedicine and Connected Healthcare Center of China, as of June 2021, there were 239 million users accessing health care services online and more than 1600 internet-based hospitals in China [31]. Another survey revealed that approximately 63% (n=465) of the surveyed hospitals (738 hospitals across 30 provinces) used cloud services to some extent in 2022 [32]. Moreover, the COVID-19 pandemic has further fueled the demand for online health care services [6]. Consequently, these services have become as commonplace in our lives as online shopping.

##### Older Adult Care: The Older Adult Care Industry Urgently Needs Advanced Technological Support

By the end of November 2020, China’s population of people aged 60 years and older was 264,018,766, accounting for 19% of the total population, making it the country with both the largest older population and the fastest-aging society worldwide [33]. Consequently, relying solely on their children, nursing homes, or communities to provide older adult care services has become increasingly impractical. Recognizing this challenge, the National Health Commission of China issued a document in October 2019 emphasizing the need to fully harness modern technologies such as cloud computing, artificial intelligence (AI), and the Internet of Things to develop an intelligent service model known as “Internet plus Healthy Aging” [34].

#### External Threats (T)

##### Market: Technological Monopolies Pose a Significant Threat to the Digital Autonomy of Nations

A total of 81 (81%) of the Forbes Top 100 cloud computing companies are American [35], and they possess significant technological and capital advantages. They continuously expand their global market share; they captured 60%-70% (JPY ¥1,725-¥2,012 billion [US $11.6-$13.6 million]) of the Japanese cloud market in 2020 [36] and 69% of the European cloud market in 2021 [37]. The passage of the Clarifying Lawful Use of Data Abroad Act (CLOUD Act) by the US Congress in March 2018 caused European countries to feel threatened due to the potential loss of digital sovereignty [15], which prompted them to initiate their “European cloud” project in 2020 [27]. Other nations may face similar challenges.

##### Competition: Fierce Competition May Lead to Uncertain Levels of Service Quality

In November 2021, 5 bidders for a public cloud service project in Shijiazhuang City, China, submitted bids of CNY ¥0, sparking concerns among stakeholders [38]. The intense competition may lead to unpredictable service performance issues for users, such as limiting hospitals’ access to better pricing and a wider range of choices by restricting the interoperability and portability of HISs or causing sudden disruptions in cloud-based medical services after winning the bid, which poses significant risks to patient safety [16].

##### Security: Hospitals Express Apprehensions Regarding Diverse Cyber-Attacks Targeting Cloud Infrastructure

Security is the primary challenge for cloud-based systems due to various cyberattacks faced by current cloud environments, especially in the health care sector where sensitive data such as personal privacy, health records, diagnostics, and treatments are stored [13]. Even prominent cloud providers like Azure (Microsoft), Docker Hub (Docker), and Everis (NTT DATA) have experienced malicious intrusions [30], while both the United Kingdom’s National Health Service (NHS) in 2017 and Ireland’s Department of Health information system in 2021 were both targeted and resulted in a complete paralysis [39,40].

##### Legislation: The Lack of Precise Legislation Hinders the Efficient Implementation and Enforcement of Regulatory Measures

To support the implementation of the “Cloud Normal” and “Internet Plus” strategies, the Chinese government has enacted laws, regulations, and management measures. However, there are limited directly applicable legal provisions for cloud migration of HISs. Imperfect laws and regulations, insufficient safety standards, unclear legal liabilities, and the absence of a damage assessment mechanism hinder the proper development of cloud services. As a result, doctors and patients may encounter challenges in protecting their rights during disputes [17].

#### Internal Strengths (S)

##### Data: Hospitals Generate Substantial Volumes of Medical Data on a Daily Basis

Hospitals are natural suppliers of big data. For instance, the Chinese National Cloud-Based Telepathology System (CNCTPS) has collected 23,167 cases and served 9240 users in 4 years (2016-2019), providing comprehensive details from whole-slide images to diagnostic reports [5]. Additionally, medical big data can provide substantial value to both hospitals and patients. For example, the aforementioned CNCTPS application can save patients around US $300,000 per year [5]. Abundant electronic health and care records in the United Kingdom’s NHS can reduce hospital operational costs by approximately £5 billion (US $ 3.9 billion) annually and save patient welfare expenses by roughly £4.6 billion (US $3.6 billion) [41]. Furthermore, traditional computing tools are unable to handle the processing and analysis of such massive amounts of data—this is exactly where cloud computing technology excels [18].

##### Business: The Provision of Comprehensive Medical Services Necessitates Extensive Interdisciplinary Collaboration

Medical services are complex and innovative, requiring synchronization of knowledge, technology, experience, and resources from diverse disciplines. Cloud computing provides extensive connectivity, offering robust support for these tasks, including interdisciplinary expert consultations, collaborative surgeries, and integrating medicine and care [19]. The Huashan Hospital, affiliated with Fudan University, uses a medical consortium cloud platform where experts from higher-level hospitals offer diagnostic advice to lower-level hospitals for subsequent care and daily treatment, ensuring positive outcomes for patients with epilepsy [42].

##### Application: Multiple Cloud Technology Applications Have Been Effectively Implemented Across Various Medical Domains

As shown in Figure 2, cloud technology is receiving increasingly extensive research and application in the medical field, and even some regional or medical alliances have constructed their own medical cloud platforms to store health data, share medical images, and facilitate collaboration. Furthermore, a national survey conducted in 2022 also confirmed these findings by revealing that out of the 738 surveyed hospitals, 63% (n=465) partially used cloud services across nearly 20 different medical business scenarios [32]. These effective practices can serve as valuable references and support for other hospitals yet to implement such initiatives.

##### Demand: The Provision of Health-Centered Medical Services Necessitates Advanced Technological Support

The transition from disease-centered to health-centered hospital development in the new era has rendered traditional HISs increasingly inadequate as they were previously designed solely for managing information within hospitals. Cloud computing can significantly expand hospitals’ medical services beyond their physical premises, enabling online chronic disease management, individual life-cycle health surveillance, and remote diagnosis for patients in remote areas. This enhancement empowers hospitals to provide health-centered medical services [20]. The findings of this study also strongly support this notion. As depicted in Figure 3, cloud technology has been extensively used in closely associated domains with patients, encompassing disease monitoring, health surveillance, clinical diagnosis and treatment, and safe medication tracking.

#### Internal Weaknesses (W)

##### System: The Complexity of HISs Poses Challenges for Hospitals When Migrating Them to the Cloud

The HISs are the most complex organizational information management systems developed by various contractors in diverse environments, covering a wide range of business functions and user groups [21], as depicted in Figure 3, with only 94 articles included but spanning across 22 distinct disciplinary domains as well. Therefore, the cloud migration of HISs presents significant challenges, particularly for those systems abandoned by development companies due to insolvency or insufficient technical support. Nevertheless, if there existed an all-encompassing and authoritative medical cloud platform enabling hospitals to tailor services based on their specific requirements, it would undoubtedly expedite the overall migration process.

##### Security: The Security of Existing Hospital Networks Still Faces Numerous Risks

Currently, most HISs still operate in self-constructed networks instead of using cloud-based solutions, which poses information security challenges due to insufficient infrastructure, overreliance on a single protective measure, incomplete regulatory frameworks, and potential vulnerabilities from privilege abuse [22]. For example, the 2019-2020 China Hospital Informatization Survey Report revealed that around 28% (n=282) of surveyed hospitals experienced unplanned core system failures lasting more than 30 minutes [43]. To effectively address these concerns, proficient IT teams like reputable cloud vendors or organizations equipped with advanced technologies such as cloud computing should collaborate rather than solely rely on in-house hospital IT capabilities.

##### Plan: Strategic Planning and Resource Allocation in Hospitals Exhibit Certain Deficiencies

According to Figure 3, more than half of the research articles focused on hospital-specific systems for various tasks. These systems still adhered to traditional information system designs, had limited scalability and functionality, and operated independently. As a result, there were significant challenges in effectively using cloud computing’s computing capabilities, storage capacity, and integrated analysis to generate valuable information supporting government scientific decision-making. The survey results from China’s National Health Commission also confirmed this point as many internet hospitals were not fully developed yet and encountered diverse issues [44]. Moreover, only 14% (n=101) of hospitals had migrated their core business operations to the cloud with a mere 13% (n=100) planning to do so in the next 3-5 years [32]. Therefore, it is crucial for hospitals to reorganize and integrate their operations and resources before incorporating their HISs into the cloud in order to meet the demands of cloud capabilities and new health care models.

##### Personnel: The Allocation of Information Personnel Is Inadequate and Lacks Specialization Levels

With the increasing integration of cloud computing, AI, and robotics, hospitals urgently require highly skilled IT professionals to effectively implement these new technologies [23]. However, a national survey in 2021 revealed that the average number of information department personnel in 9376 secondary and tertiary hospitals was only 6. Most of these personnel held undergraduate or junior college computer degrees and possessed limited interdisciplinary expertise. This falls significantly below national standards [24], particularly for hospitals below grade 2 or in economically underdeveloped areas [45].

##### Investment: Primary Hospitals Lack Sufficient Investment in Information Technology and Cloud Services

According to a 2020 survey by the National Health Commission of China, most primary hospitals allocated less than 1% of their budgets to HIS development, facing challenges such as unstable funding and support [25]. A nationwide survey conducted in 2022 revealed that only 53% of the surveyed 738 hospitals had expenses related to public cloud services in the previous 2 years, with 54% spending less than US $14,000. Particularly for primary hospitals, establishing a cloud service system is even more financially challenging [32]. Clearly, these primary hospitals require more reliable financial guarantees for the smooth operation of their HISs and cloud services.

## Discussion

### Principal Findings

**Figure 4:**
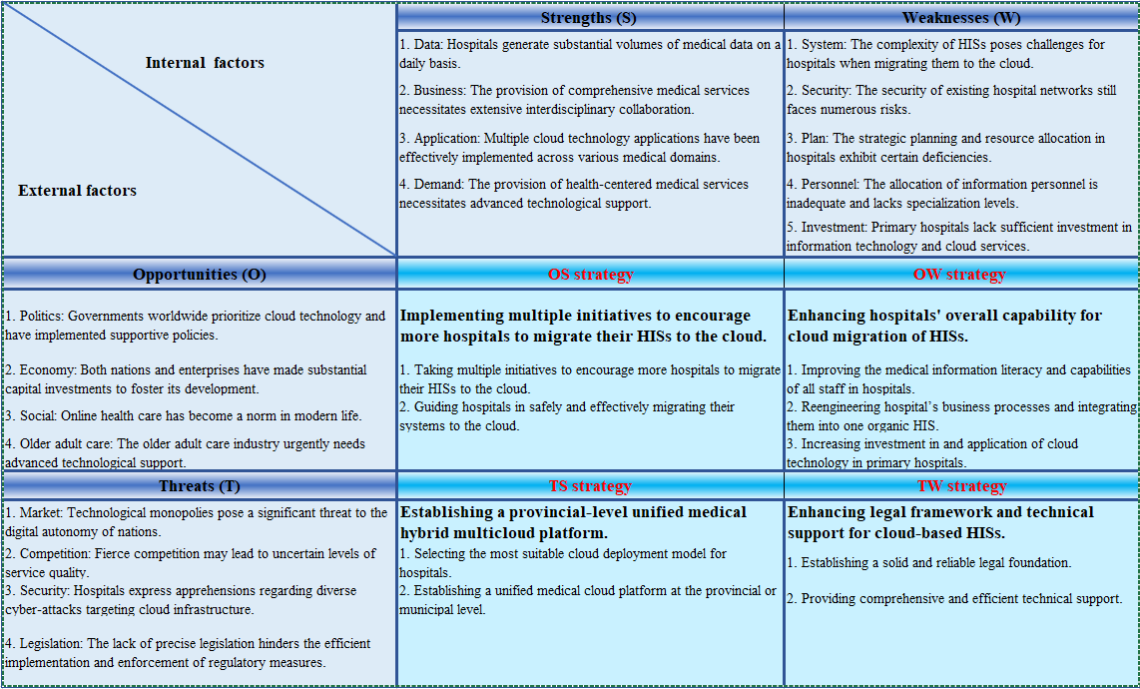
SWOT analysis and response strategies diagram for cloud migration of HISs. HIS: hospital information system; SWOT: Strengths, Weaknesses, Opportunities, and Threats.

Extensive literature review and systematic SWOT analysis indicate that cloud computing is increasingly being applied in nearly 22 discipline domains in Chinese hospitals; it plays a crucial role in monitoring patient-related diseases, health surveillance, clinical diagnosis and treatment, and safe medication tracking. However, more than half of the research and applications are limited to cloud-based systems for specific hospital tasks, which fail to fully leverage the robust integrated analytical capabilities of cloud computing due to limited data scale and functionality that could otherwise generate valuable information supporting hospital or government decision-making processes. Additionally, challenges such as market sovereignty disputes, intense industry competition, network attacks, inadequate regulation, and hospitals’ internal weaknesses like complexity of HISs, insufficient resource integration, and limited manpower and investment, hinder widespread adoption of cloud technology among most hospitals that exhibit a relatively weak willingness to migrate their core operations to the cloud within the next 3-5 years. Nevertheless, cloud computing is widely recognized as a novel infrastructure driving global economic growth. Integrating cloud technology in hospitals can enhance medical service quality, foster interdisciplinary collaboration and remote consultations, and promote coordinated development within regional health care economies. Consequently, it is imperative for hospitals and health authorities to pay special attention to this matter and actively implement diverse strategies to facilitate its advancement. Based on the aforementioned research findings, this study proposes a set of promotional strategies for collective deliberation among peers. The overall framework depicting these proposed strategies is illustrated in Figure 4, which will be further elucidated in subsequent sections.

### Implementing Multiple Initiatives to Encourage More Hospitals to Migrate Their HISs to the Cloud

The primary objective of this strategy is to address the issue of “whether or not to adopt cloud technology.” Based on the outcomes of the SWOT analysis, despite the pressing need for cloud technology to enhance health-centered medical service delivery today, hospitals remain cautious about its implementation due to external threats and internal weaknesses. Furthermore, providing cloud-based medical services has brought about a significant transformation within the medical industry that exceeds traditional independent operations and self-financing models used by hospitals. Therefore, governments should make greater efforts by implementing more active measures such as policy guidance, training planning, demonstration projects, or provision of cloud vouchers, in order to encourage more hospitals to securely migrate their HISs onto the cloud and meet demands for innovative medical services in this modern era.

### Enhancing Hospitals’ Overall Capability for Cloud Migration of HISs

The strategy aims to address the issue of “what preparations are necessary.” As previously mentioned, cloud migration of HISs is a highly intricate system engineering project that requires hospitals to be fully prepared for its successful implementation. These preparations encompass various aspects including, but not limited to the following. First, human resources: hospitals should enhance employees’ medical information literacy and application skills through comprehensive training programs, specialized lectures, or successful case studies. Second, material resources: hospitals should redesign and integrate existing systems and resources based on future development, existing foundations, and expert recommendations in order to optimize the use of cloud resources for acquiring more valuable information. Third, financial resources: hospitals require long-term financial investment planning to support the provision of cloud-based medical services. Moreover, health authorities should acknowledge that primary hospitals serve as both frontline institutions addressing medical needs and significant sources of authentic data. Therefore, moderate increases in investment in HIS construction of primary hospitals are necessary to ensure a continuous input of firsthand authentic data. Fourth, tools: a hospital that has robust IT capabilities can leverage free cloud migration consultation and tools provided by major cloud providers such as Alibaba, Tencent, Google, Microsoft, and Amazon Web Services, which can expedite the process of migrating information systems. However, it should be noted that the cloud services used (eg, computing and storage) may incur charges.

### Establishing a Provincial-Level Unified Medical Hybrid Multi-Cloud Platform

The strategy aims to address the issue of “how to implement changes efficiently.” In response to numerous complex internal and external situations, this study proposes a coping strategy: establishing a unified medical hybrid multi-cloud platform in each province or municipality.

First, the hybrid multi-cloud platform highly aligns with hospital operations. Hospitals require private clouds for storing sensitive and core data, nonpublic community clouds for internal consultations and other collaborations, public clouds for providing more extensive medical services to the public, and adopting a “multi-cloud” strategy to reduce risks such as vendor lock-in or declining service quality.

Second, a medical cloud platform at the provincial or municipal level can achieve maintainable security and more highly effective cloud migration. In comparison to hospitals, health authorities at this level possess stronger technological expertise, greater manpower resources, maintainable financial support, and relevant assets for constructing comprehensive platforms while effectively mitigating internal and external risks. Moreover, this approach can also foster overall advancements in cloud migration and system function quality of hospitals (particularly primary ones), as well as minimize redundant construction and maintenance expenses.

Last but most importantly, the scale of data possessed by one or several individual hospitals is insufficient to constitute true big data, limiting the opportunities for leveraging cloud computing’s powerful intelligent analysis capabilities in uncovering hidden objective laws that can support the government to make scientific decisions. Considering factors such as data scale, cloud computing capabilities, and government economic support capacity, a provincial or municipal regional medical cloud platform is a more suitable choice.

### Enhancing Legal Framework and Technical Support for Cloud-Based HISs

The primary goal of this strategy is to address the issue of “how to create a supportive environment.” As an ancient Chinese proverb states, “A single log cannot support a crumbling building,” indicating that relying solely on a provincial-level medical cloud platform is still insufficient to counter all external threats and internal risks faced by hospitals. Therefore, it requires a more proactive role from the government, which strengthens cooperation with relevant departments and enterprises to build a more robust and secure supporting environment for medical cloud platforms. Specifically, 2 aspects of support are necessary. First, credible legal support: although the Chinese government has been improving laws regarding cybersecurity, personal information protection, and data security, its support for cloud-based medical services remains inadequate. For example, in resolving disputes related to cloud medical services, health authorities still rely on laws such as the Physician Practice Law and Regulations on Prevention and Handling of Medical Disputes in China. However, these regulations have not explicitly defined the status and responsibilities of all parties involved in cloud-based medical services, which poses challenges in terms of judgments and penalties [26]. Therefore, it is essential to further refine relevant legislation and update existing regulations regarding doctors’ practice rights, insurance responsibility, and reimbursement for medical insurance, ensuring doctors and patients can confidently participate in cloud-based medical services. Second, holistic technical support: as indicated by SWOT analysis results, hospitals often lack professionals with deep knowledge of cutting-edge technologies like cloud computing, AI, and big data. Therefore, establishing an organization like a medical cloud technology association becomes necessary. This organization should consist of experts from various relevant fields, including IT, communication, engineering, cryptography, medicine, and more. Their responsibilities would include devising unified long-term plans and action plans, standardizing contracts between hospitals and cloud service providers, reviewing hospitals’ cloud migration plans and contracts, and conducting regular evaluations of the construction and operation of cloud-based HISs.

### Conclusions

In conclusion, cloud computing is prioritized by the Chinese government as a “national strategic emerging industry.” Despite encountering numerous challenges, the cloud migration of HISs in China exemplifies a prevailing development trend. Therefore, hospitals should adopt an open mindset and focus on enhancing their capabilities to develop medical services based on the cloud. Health authorities should also use more effective strategies to incentivize hospitals to migrate their HISs safely to the cloud, thereby fostering the flourishing growth of a novel health-centered medical industry.

The main contribution of this study is a comprehensive literature review and systematic SWOT analysis on the current status of cloud migration of HISs in China, and corresponding strategies for different combinations of internal and external influencing factors. It can help hospitals gain a clearer understanding of the overall situation while having more specific goals and methods when planning and implementing related work. Moreover, it can serve as a foundation for health authorities to develop policies aligned with the development of hospital informatization in the new era, as well as provide references for other countries or regions facing similar challenges.

There are 2 limitations in this study: first, not all personnel from hospitals contribute to writing papers, thus limiting the comprehensiveness of literature information; second, the SWOT analysis method assumes a distinction between internal and external factors, as well as a differentiation between strengths and weaknesses, overlooking the interrelated effects among influencing factors. To supplement and improve these aspects, more empirical investigation data are needed, along with a more rigorous analysis of the interactions among influencing factors. This will be the focus of my future research.

## Appendix

Multimedia Appendix 1 Academic paper database.

Multimedia Appendix 2 Supplementary database.

## Abbreviations

AI: artificial intelligence
CAGR: compound annual growth rate
CLOUD Act: Clarifying Lawful Use of Data Abroad Act
CNCTPS: Chinese National Cloud-Based Telepathology System
DOI: digital object identifier
HIS: hospital information system
NHS: National Health Service
PMID: PubMed unique identifier
SWOT: Strengths, Weaknesses, Opportunities, and Threats

## Data Availability

The data sets generated and analyzed during this study are not publicly available but are available from the corresponding author on reasonable request.
